# Supplementary material for: Asynchronous magnetic resonance elastography: Shear wave speed reconstruction using noise correlation of incoherent waves
Source: Magn Reson Med. 2022 Oct 27;89(3):990–1001. doi: 10.1002/mrm.29502 (PMC9792433; doi:10.1002/mrm.29502)
Supplement: Supplementary file 1 — DATA S1 MATLAB code used to generate the numerical simulations of Figure 1 [file MRM-89-990-s002.zip › k-Wave/helpfiles/example_pr_2D_tr_bandlimited_sensors.html]

Image Reconstruction With Bandlimited Sensors Example (k-Wave)


# Image Reconstruction With Bandlimited Sensors Example

This example demonstrates how the bandwidth of sensor elements can give rise to artefacts in time reversal photoacoustic image reconstruction.
It builds on the previous 2D time reversal examples.

- Open the file in the MATLAB Editor
- Run the file in MATLAB

## Contents

- Running the simulation and image reconstruction
- Applying a high-pass filter
- Applying a low-pass filter
- Modelling the sensor frequency response

## Running the simulation and image reconstruction

The sensor data is simulated using `kspaceFirstOrder2D` in the same way as previous examples. In this example, the initial pressure distribution is set to be three disks created using `makeDisc` with different magnitudes and diameters. A continuous circular binary sensor mask created using `makeCircle` is used to avoid additional limited view artifacts. The smoothed initial pressure distribution is reproduced after time reversal image reconstruction by passing the simulated sensor data directly to `sensor.time_reversal_boundary_data` (in this case committing the inverse crime in which computations are run forwards and backwards using the same parameters).


## Applying a high-pass filter

To illustrate the effect of removing the low frequency data, a high pass filter is applied to the simulated sensor data using `applyFilter`. By default, this uses a causal FIR filter designed using the Kaiser windowing method. The causal nature of the filter means that the temporal signals will become offset from their original position. This offset is avoided by using a zero phase filter by setting the optional input `'ZeroPhase'` to `true`. The filter type is set to `'HighPass'` and the cutoff frequency to 1 MHz.

```
% filter the sensor data using a high pass filter
Fs = 1/kgrid.dt;        % [Hz]
cutoff_freq = 1e6;      % [Hz]
sensor_data_high_pass = zeros(size(sensor_data));
for index = 1:sum(sensor.mask(:))
    sensor_data_high_pass(index, :) = applyFilter(sensor_data(index, :), Fs, cutoff_freq, 'HighPass', 'ZeroPhase', true);
end
```

If the initial pressure is reconstructed using the filtered sensor data, only the edges of the discs are reconstructed. This is due to the missing low frequency data.


## Applying a low-pass filter

To illustrate the effect of removing the high frequency data, a low pass filter is applied to the simulated sensor data, again using `applyFilter`. The filter type is set to `'LowPass'`, the cutoff frequency to 1 MHz, and the optional input `'ZeroPhase'` is again set to `true`.

```
% filter the sensor data using a low pass filter
Fs = 1/kgrid.dt;        % [Hz]
cutoff_freq = 1e6;      % [Hz]
sensor_data_low_pass = zeros(size(sensor_data));
for index = 1:sum(sensor.mask(:))
    sensor_data_low_pass(index, :) = applyFilter(sensor_data(index, :), Fs, cutoff_freq, 'LowPass', 'ZeroPhase', true);
end
```

If the initial pressure is reconstructed using the filtered sensor data, the edges of the disc become blurred. This is due to the missing high frequency data.


## Modelling the sensor frequency response

The construction of many conventional ultrasound transducers results in a reduction in sensitivity at both low and high frequencies about some centre frequency. This type of response can be approximated by applying a frequency domain Gaussian filter to the recorded sensor data using the centre frequency and percentage bandwidth of the transducer (the latter corresponds to the full width at half maximum of the frequency response as a percentage of the centre frequency). This can be applied using `gaussianFilter` (this function is used by the simulation functions when `sensor.frequency_response` is defined). Here, a transducer with a centre frequency of 3 MHz and a bandwidth of 100% is modelled.

```
% filter the sensor data using a Gaussian filter
Fs = 1/kgrid.dt;        % [Hz]
center_freq = 3e6;      % [Hz]
bandwidth = 100;        % [%]
sensor_data_gaussian = gaussianFilter(sensor_data, Fs, center_freq, bandwidth);
```

If the initial pressure is reconstructed using the Gaussian filtered sensor data, again the edges of the discs are reconstructed more prominently. Because the bandwidth of this filter is quite wide, not all the low frequency data is removed and thus some of the information from the middle of the discs can still be seen.

A profile through the centre of the largest disc for the different reconstructions is shown below for comparison.
